# Supplementary material for: Past terrestrial hydroclimate sensitivity controlled by Earth system feedbacks
Source: Nat Commun. 2022 Mar 14;13:1306. doi: 10.1038/s41467-022-28814-7 (PMC8921287; doi:10.1038/s41467-022-28814-7)
Supplement: Supplementary file 1 — Supplementary Information [file 41467_2022_28814_MOESM1_ESM.pdf]

Supplementary Materials for

Past terrestrial hydroclimate sensitivity controlled by Earth System Feedbacks

R. Feng[1*], T. Bhattacharya[2], B. Otto-Bliesner[3], E. Brady[3], A. Haywood[4], J. Tindall[4], S. Hunter[4], A. Abe-Ouchi[5], W.-L. Chan[5], M. Kageyama[6], C. Contoux[6], C. Guo[7], X. Li[8], G. Lohmann[9], C. Stepanek[9], N. Tan[10], Q. Zhang[11], Z. Zhang[8], Z. Han[12], C. J. R. Williams[13], D. J. Lunt[13], H. Dowsett[14], D. Chandan[15], W. R. Peltier[15]

*Corresponding author. Email: ran.feng@uconn.edu

This PDF file includes:

Table S1
Table S2
Figs. S1 to S13
References (1 to 39)

Other Supplementary Materials for this manuscript include the following:

Data S1 Mid-Pliocene (3.0 – 3.3 Ma) hydroclimate indicators compiled from published records.

Supplementary Text

Sensitivity experiments using CESM2

We carried out two new simulations using the community Earth System Model version 2 (individual model components are Community Atmospheric Model version 6, Community Land Model version 5, Parallel Ocean Program version 2, and Community Ice CodE version 5) to quantify individual effects of elevated CO_2, changes in paleo-geography and topography, and vegetation and ice sheets on mid-Pliocene P-E changes. These new experiments separately feature Pliocene levels of carbon dioxide (400 ppm CO_2) coupled with preindustrial boundary conditions (e.g. ice sheets and vegetation and geography and topography) in the case of E400. This simulation was used to isolate the influence of Pliocene CO_2 (F_{CO2}). For Eo400, the simulation features preindustrial vegetation and ice sheets and otherwise mid-Pliocene CO_2 and boundary conditions, and can be compared to the full Pliocene simulation to isolate the influence of vegetation and ice cover (F_{vegice}). The influence of geography and topography (F_{geotop}) was isolated by subtracting E400 from Eo400.

Eo400 and E400 are initialized with ocean states and terrestrial carbon and nitrogen states from previously published runs of the mid-Pliocene, and preindustrial which feature the same geography and topography as Eo400 and E400 respectively. Each of which was run for more than 500 model years. Model equilibrium is diagnosed with global mean net top of atmosphere radiation imbalance (F_{net}) and global mean surface temperature (T_s). For the last 100 model years, global mean F_{net} of all simulations is ~ 0.2W/m^2 for both simulations, and trends of global mean T_s are 0.1 and 0.2°C per century (Fig. S9).

Proxy recorded Pliocene regional hydroclimate patterns

We compiled Pliocene hydroclimate indicators from published records (Data S1). We rely on the author's original interpretations about whether the record reflects, on average, wetter or drier conditions, or no change in hydroclimate during the mid-Pliocene compared to late Quaternary/modern conditions. Below, we provide a review of published studies on broad regional trends evident in the proxy records.

Supplemental Table 1 Broad regional trends of mid-Pliocene hydroclimate changes recorded by a compilation of proxy data.

Regions	Summary of published mid-Pliocene hydroclimate changes
Europe and the Mediterranean	Evidence of wetter mid-Pliocene conditions in Europe and the Mediterranean primarily come from sedimentological evidence of expanded lacustrine environments and palynological indicators of more mesic vegetation. For instance, pollen and macrobotanical remains from the lagerstatte deposit at Willershausen in modern Germany provide evidence of a slightly wetter climate[1] and pollen records[2] document mesic vegetation across the Iberian Peninsula and north Africa. Evidence from the Dacian Basin records an interval of high salinity at 3 Ma towards the end of the mid-Pliocene, which could reflect changes to the regional water budget, but has been interpreted to reflect higher water levels in the Black Sea that slightly postdate the mid-Pliocene[3]. This coheres with the

	interpretations of the Ref[4], which suggest a decrease in winter precipitation in midlatitude Europe between 4 and 3 Ma.
Africa and the Middle East	Continuous Plio-Pleistocene records of dust flux from off the coast of west Africa have been interpreted as evidence of long-term drying[5,6]. Several records also provide evidence of the expansion of ecosystems dominated by C4 plant species[7-10]. Like pollen records, the interpretation of these records is complex since these ecosystem shifts may reflect hydroclimate, but these may also be driven by changes in pCO_2 or fire regimes. However, records of stable isotopes of oxygen and hydrogen in organic and inorganic materials, which have been interpreted as reflecting an 'amount effect,' whereby higher rainfall rates result in a more depleted isotopic signature, reflect a wetter mid-Pliocene[7,11,12]. Pollen data , dust flux records, and sedimentological indicators also show wetter conditions in the Levant and Arabian Peninsula during the mid-Pliocene[13,14]. This is corroborated by a recently published stable isotopic record of hydroclimate from a cave in the Negev Desert[15].
South and East Asia	Evidence of wetter mid-Pliocene conditions in South and East Asia are primarily drawn from palynological transfer functions or faunal remains[16-18]. However, in many regions these inferences are corroborated by other indicators[19]. In the Qaidam Basin (northeastern Tibetan plateau), and in southwest China's Yuanmou region, sedimentological indicators provide evidence of a wetter mid-Pliocene in regions where pollen evidence suggests little change[14,20-23]. In the Loess Plateau region, multiple proxies provide evidence of no change or drier conditions at the mid-Pliocene compared to the Pleistocene[24,25]. However, evidence from mapping the total extent of loess deposits at different intervals during the Neogene and Quaternary provides evidence of a more mesic climate during the mid-Pliocene[26].

Supplemental Table 2 Models, references, and vegetation boundary conditions of PlioMIP2 simulations analyzed in this study.

	Modeling Group	Model reference	Vegetation
CCSM4	National Center for Atmospheric Research	27	Prescribed according to PRISM4[28]
CESM1.2	National Center for Atmospheric Research	29	Prescribed according to PRISM4[28]
CESM2	National Center for Atmospheric Research	30	Prescribed according to PRISM4[28]
COSMOS	Alfred Wegener Institute, Germany	31	Dynamic
EC-Earth 3.3	Stockholm University, Sweden	32	Prescribed according to PRISM4[28]
HadCM3	University of Leeds, UK	33	Prescribed according to PRISM4[28]
IPSL-CM5	Laboratoire des Sciences du Climat et de l'Environnement (LSCE), France	34	Prescribed according to PRISM4[28]
IPSL-CM5A2	Laboratoire des Sciences du Climat et de l'Environnement (LSCE), France	34	Prescribed according to PRISM4[28]
IPSL-CM6	Laboratoire des Sciences du Climat et de l'Environnement (LSCE), France	35	Prescribed according to PRISM4[28]
MIROC4m	Center for Climate System Research (Uni. Tokyo), JAMSTEC	36	Prescribed according to PRISM4[28]
NorESM-1L	Norwegian Research Centre, Bjerknes Centre	37	Prescribed according to PRISM4[28]
HadGEM3	UK Met Office	38	Prescribed according to PRISM4[28]
GISS-E2-1G	NASA Goddard Institute for Space Studies	39	Prescribed according to PRISM4[28]

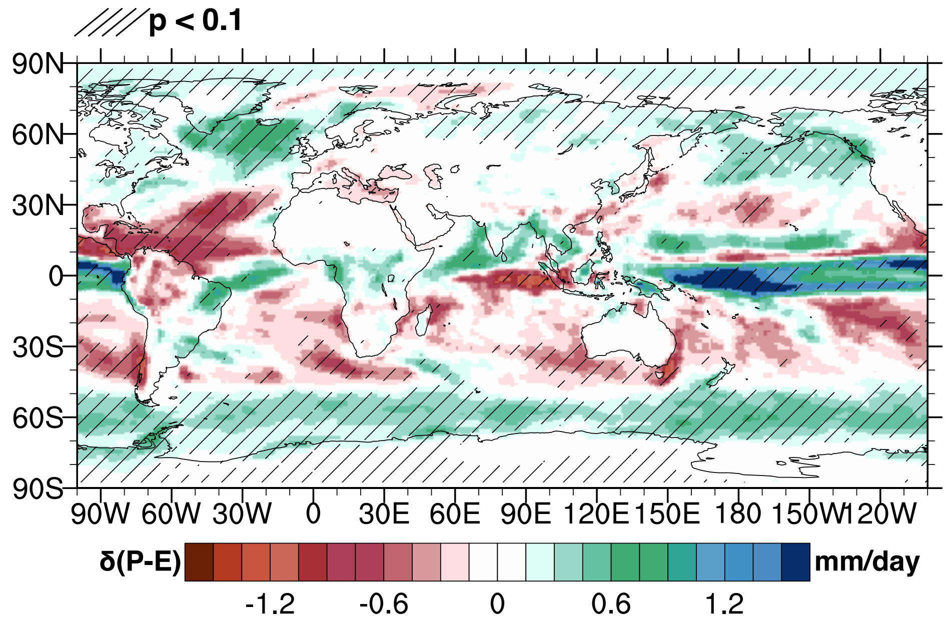

Fig. S1. δ(P-E) between mean (P-E) of 2081 to 2100 following Shared Socioeconomical Pathway 2-4.5 and year 1850 preindustrial control (20-yr average) simulated by the same set of CMIP6 models shown in Fig. 1. Hatched areas show statistically significant differences identified by Welch's t-test ($p<0.1$).

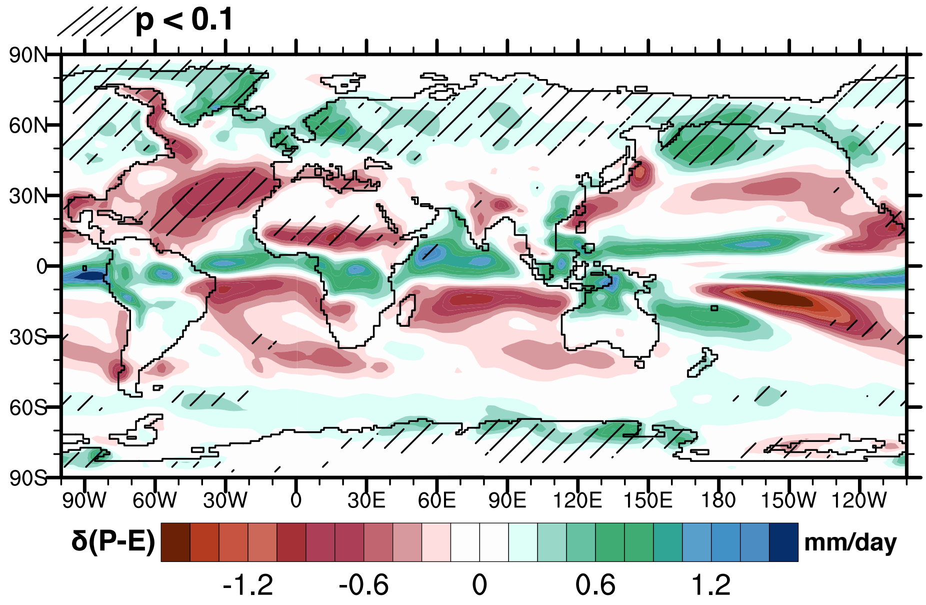

Fig. S2. Simulated terrestrial hydroclimate change between PlioMIP2 simulations and PI, referred to as δ(P-E), during the boreal winter (December to March).

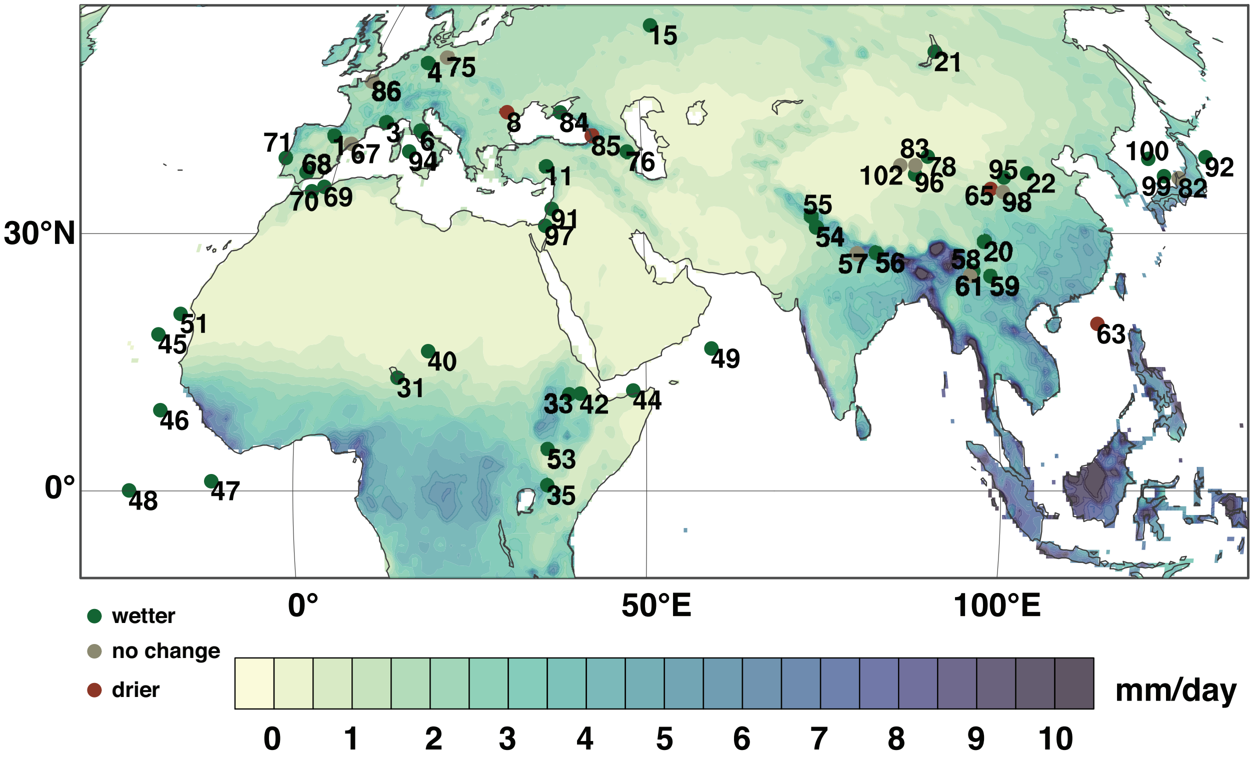

Fig. S3. Locations of sites used in our proxy compilation, set against a background of annual average rainfall rate from the GPCC. Co-located sites were combined if they were less than 150 km apart and featured same-signed anomalies. These include: Site 35, 72; 77, 22; 79,61; 65, 66; and 61,79,58,191. Site numbers correspond to site indices in supplemental file (SI_pliocene_hydroclimate.xlsx), which also contains information on proxies, chronology, and references. Note that numbers are non-continuous, since some sites in our original compilation were excluded by our quality control standards for proxy data. Original references for each site are included in the main text Methods section.

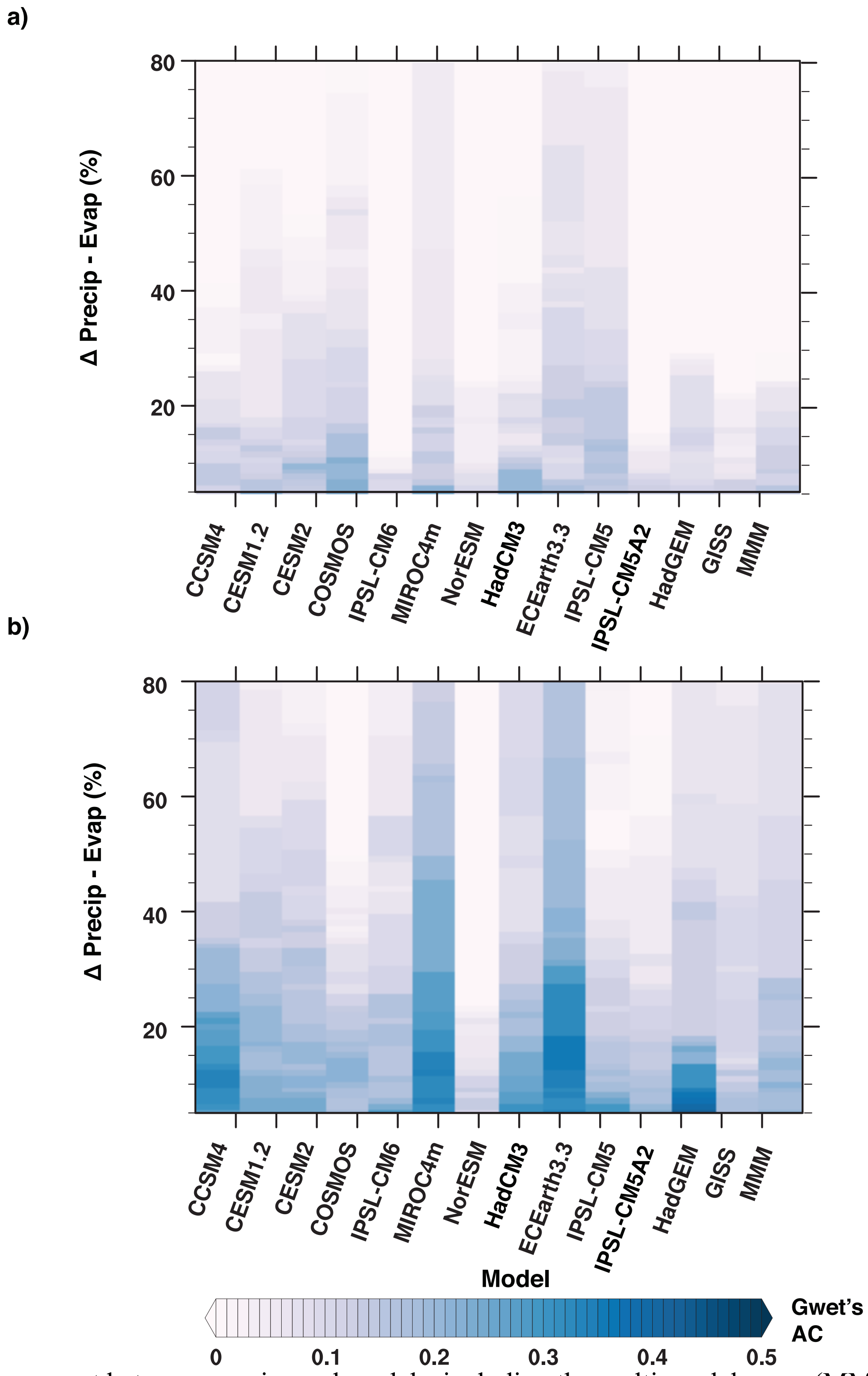

Fig. S4 Agreement between proxies and models, including the multi-model mean (MMM), in different seasons. (a) shows Gwet's AC value at different thresholds of % change in P-E for winter (December-March) rainfall only, and b) shows Gwet's AC values for summer rainfall only (July-September).

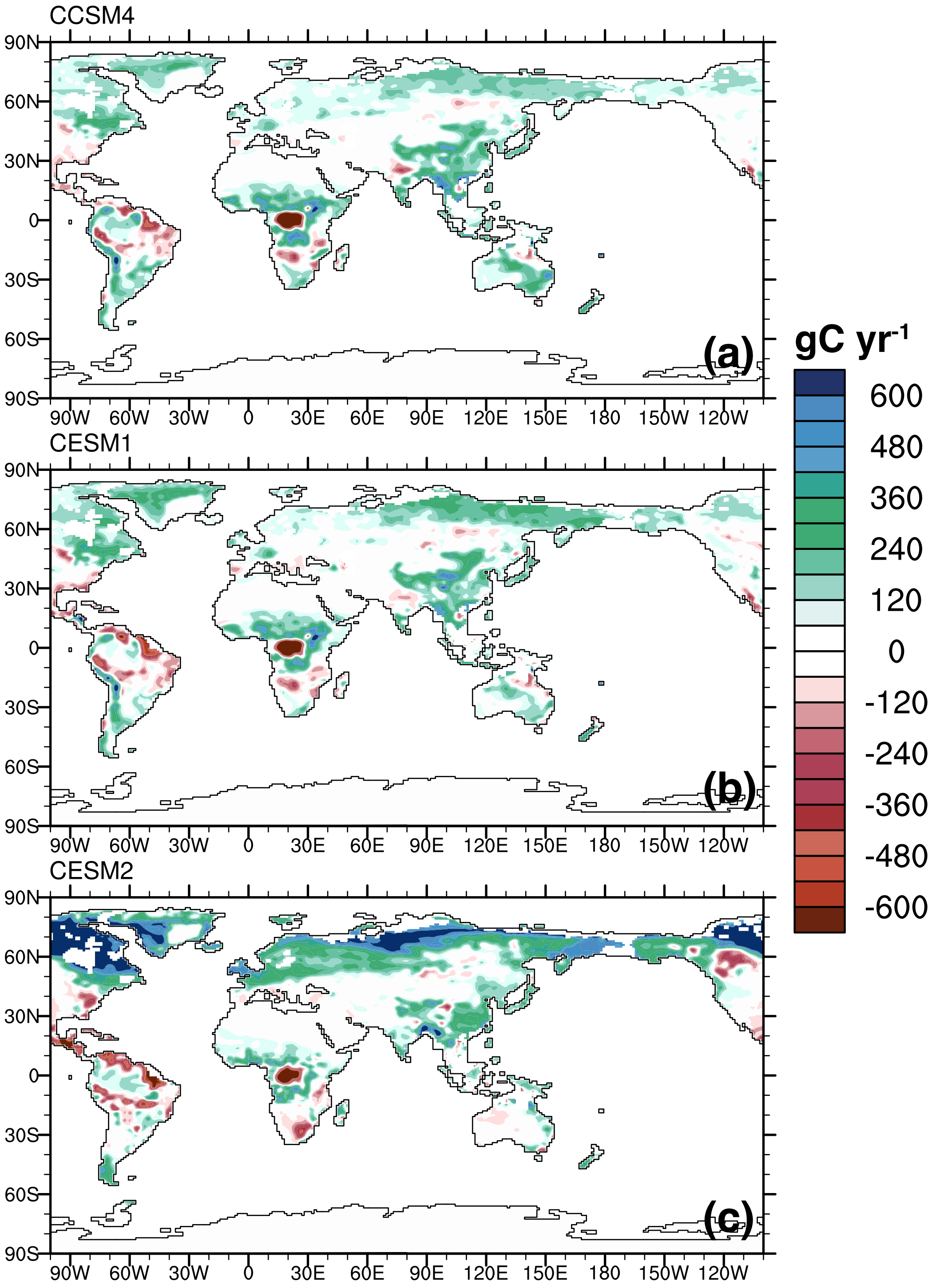

Fig. S5 Simulated changes in net primary productivity between mid-Pliocene and preindustrial by (a) Community Climate Model version 4 (CCSM4), (b) Community Earth System Model version 1 (CESM1) and (c) version 2 (CESM2).

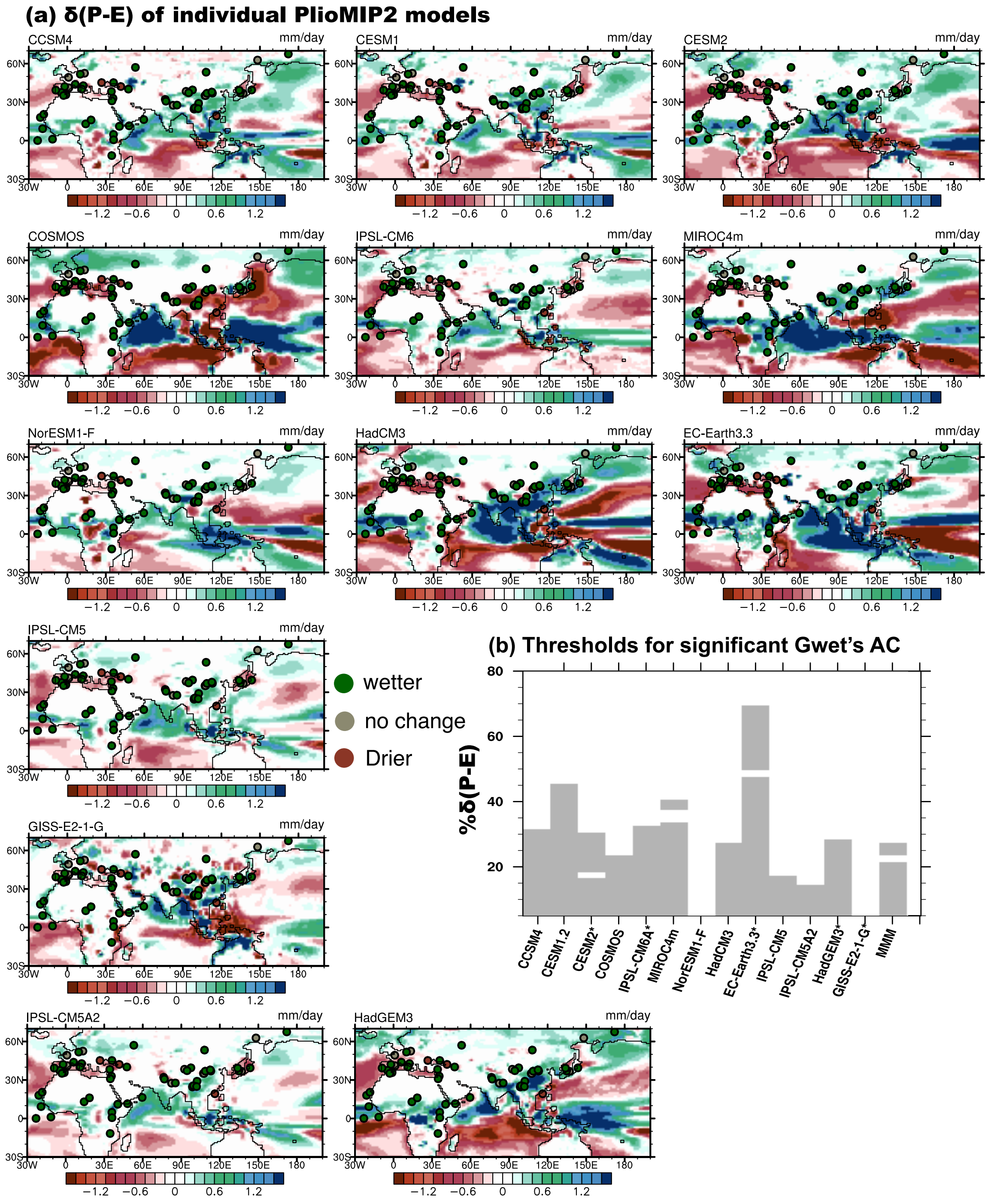

Fig. S6. (a) Change in annual mean precipitation minus evaporation (δ(P-E)) in individual PlioMIP2 models relative to preindustrial. Model name is given in the in top left of each panel, and proxy records are overlaid to show whether a given record shows wetter, drier, or no change. (b) Range of significance thresholds of %δ(P-E) at which the agreement between proxies and models shown in Figure 2 in the main text is significant, with gray colors indicating significant values of Gwet's AC at that percentage threshold.

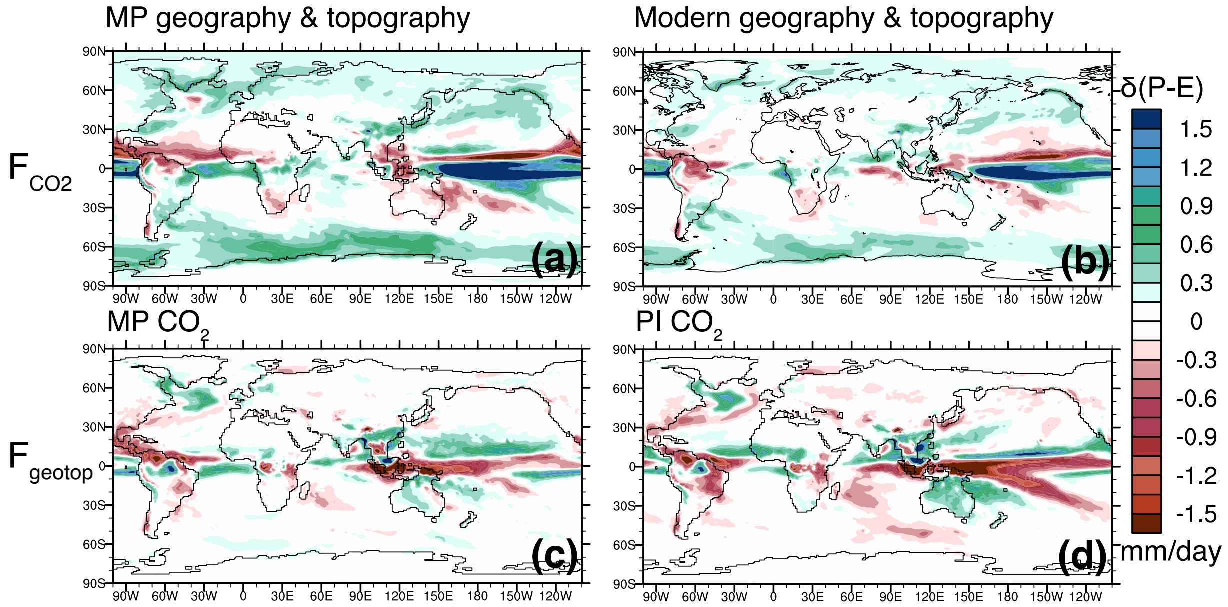

Fig. S7 Annual mean δ(P-E) responses to F_{CO2} with a) mid-Pliocene and b) PI geography and topography, and δ(P-E) responses to F_{geotop} with a) mid-Pliocene and b) PI CO_2.

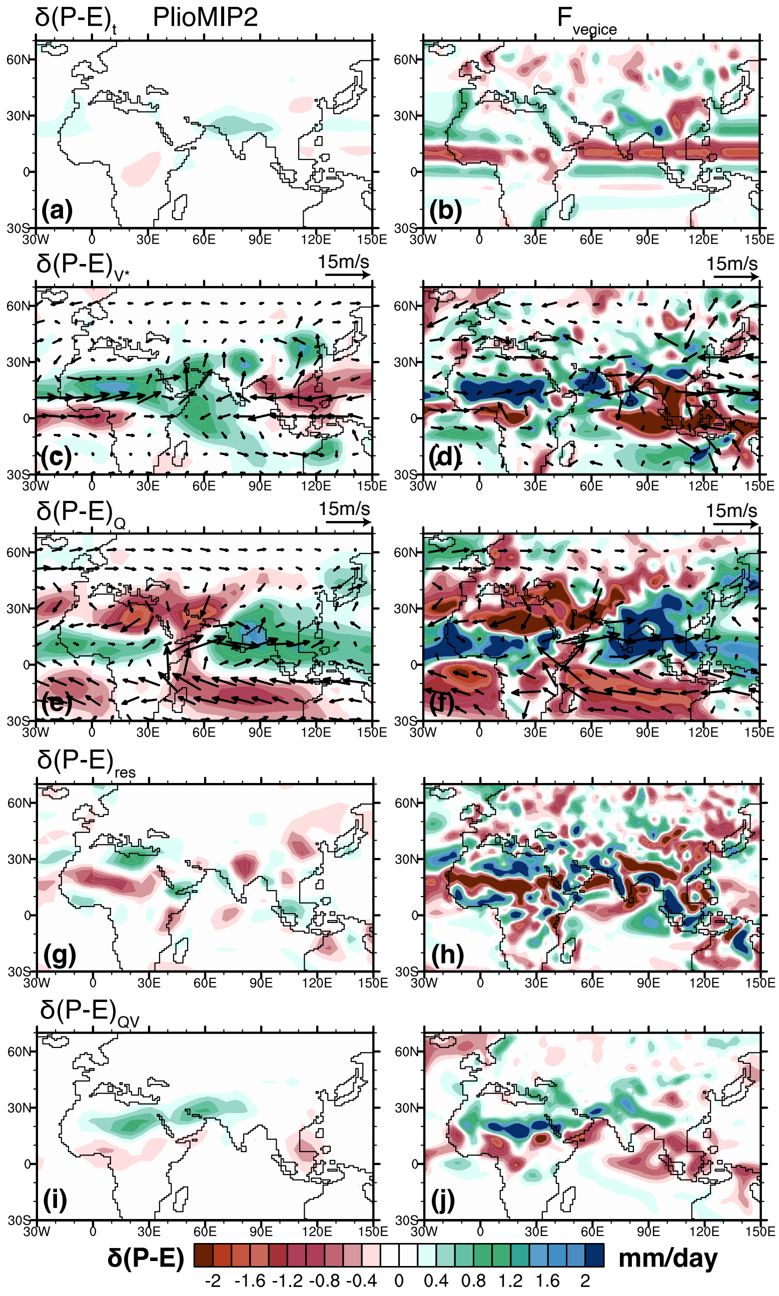

Fig. S8 June to September contributions to δ(P-E) from (a) and (b) change in the seasonal cycle ($\delta(P\text{-}E)_t$), (c) and (d) stationary wave dynamics ($\delta(P\text{-}E)_{V*}$), e) and f) tropospheric moistening ($\delta(P\text{-}E)_Q$), (g) and (h) residual combining the effect of transient eddies and topographic changes ($\delta(P\text{-}E)_{res}$), (i) and (j) covarying humidity and winds ($\delta(P\text{-}E)_{VQ}$). Left column: δ(P-E) due to Pliocene full forcing conditions estimated with PlioMIP2 MMM. Right column: δ(P-E) due to vegetation and ice sheet changes estimated with CESM2.

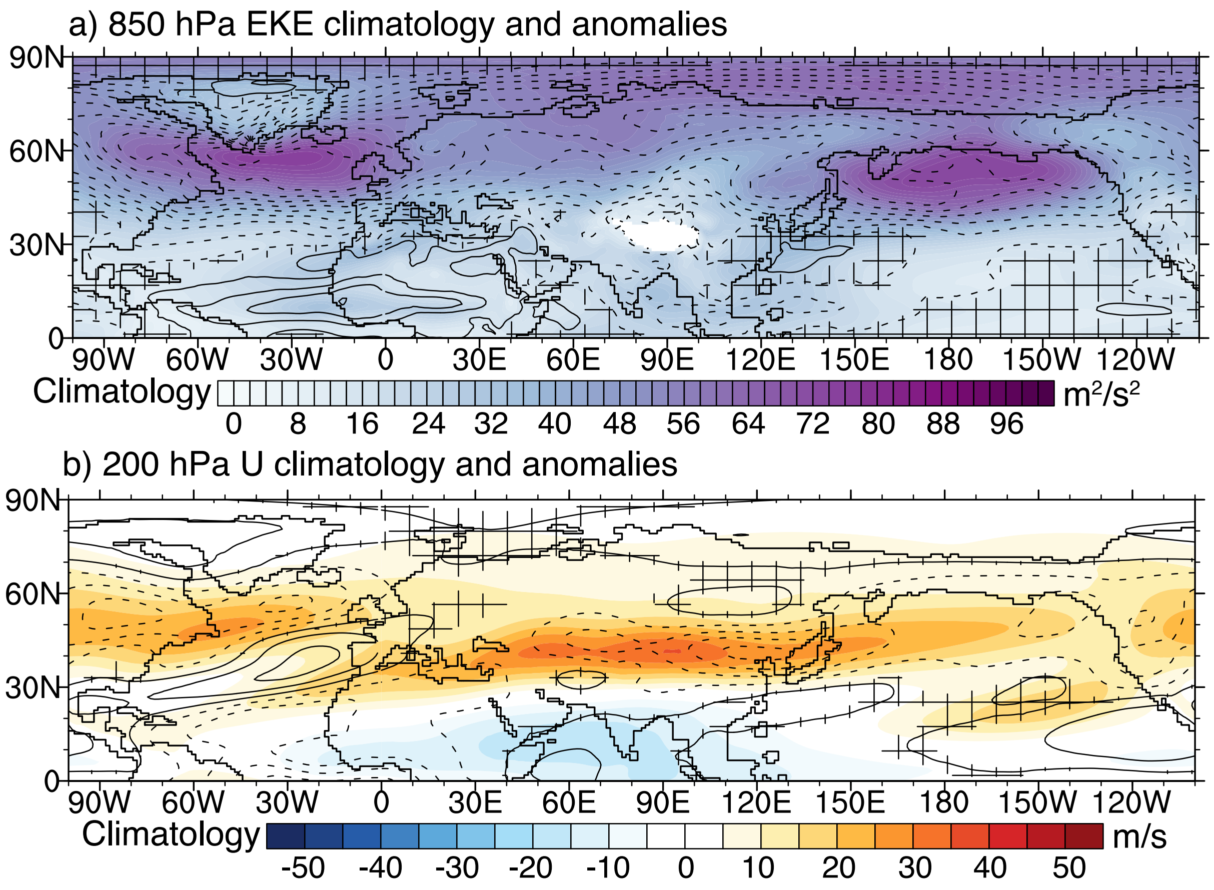

Fig. S9 Climatology of preindustrial (shaded) and changes (contour, dashed: negative; solid: positive) of a) 850 hPa eddy kinetic energy and b) 200 hPa zonal wind between PlioMIP2 and PI averaged for CCSM4, CESM1, and CESM2. Hatches show at least one model with insignificant changes compared to the 100 model year interannual variability through Student's t-test with $p>0.1$.

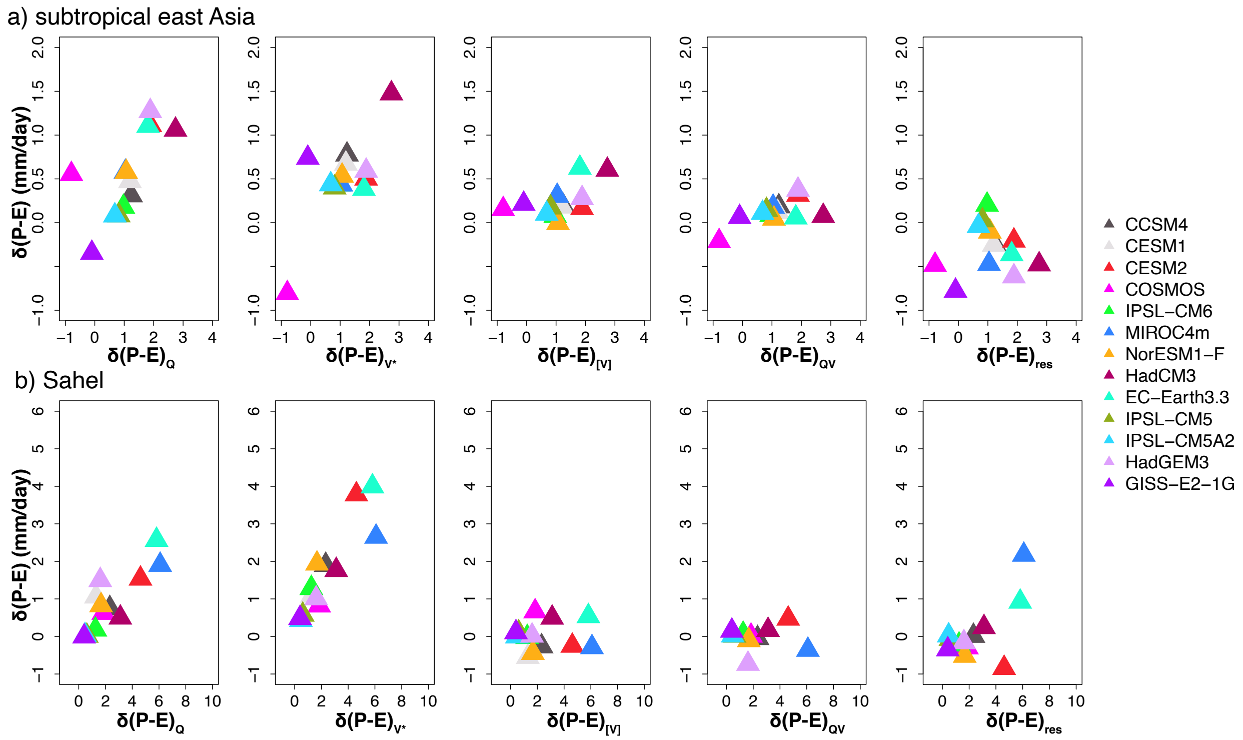

Fig. S10
δ(P-E) across a) Sahel and b) subtropical east Asia (red boxes in Fig. 4) of individual PlioMIP2 simulations as a function of changes in tropospheric humidity ($\delta(P\text{-}E)_Q$), stationary wave dynamics ($\delta(P\text{-}E)_{V*}$), zonal mean circulation ($\delta(P\text{-}E)_{[V]}$), non-linear combination of tropospheric humidity and winds ($\delta(P\text{-}E)_{QV}$), and a residual term ($\delta(P\text{-}E)_{res}$).

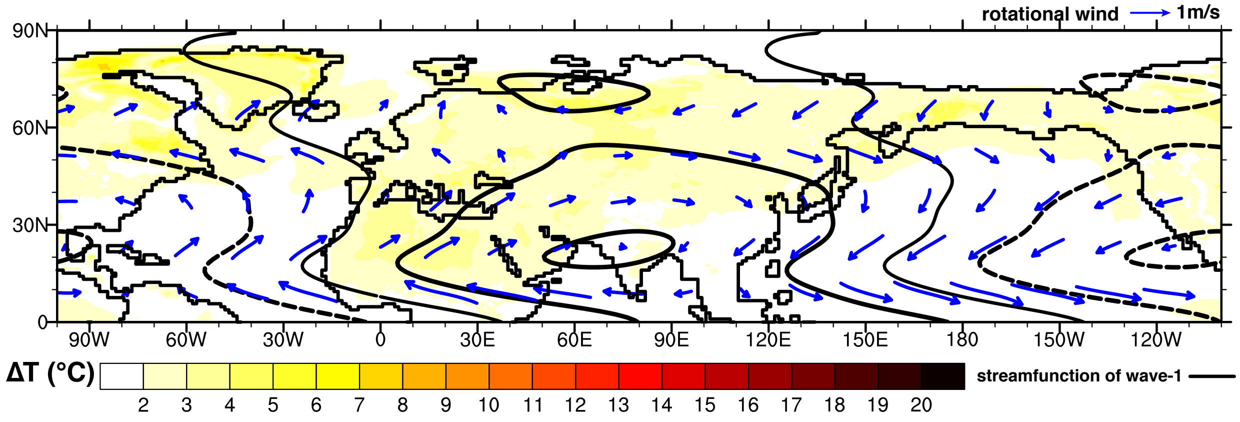

Fig. S11 Changes of surface temperature (color shaded), wave number 1 (contour), and rotational winds (vectors) of stationary wave in response to F_{CO2} simulated by CESM2. Notice that the stationary wave pattern has little dependency on surface temperature changes, but mainly reflects the high-pressure system above Tibet developed during the boreal summer.

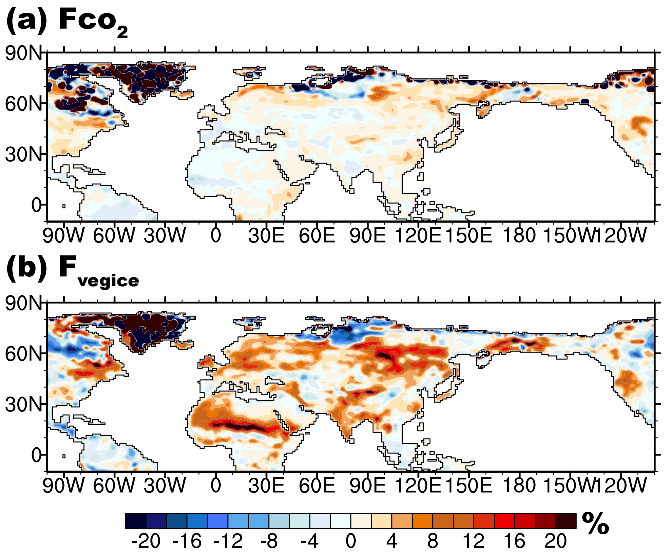

Fig. S12 Change in the % of latent heat flux in total surface heat flux (the sum of latent and sensible heat flux) due to Pliocene (a) CO_2 and (b) vegetation and ice sheet changes.

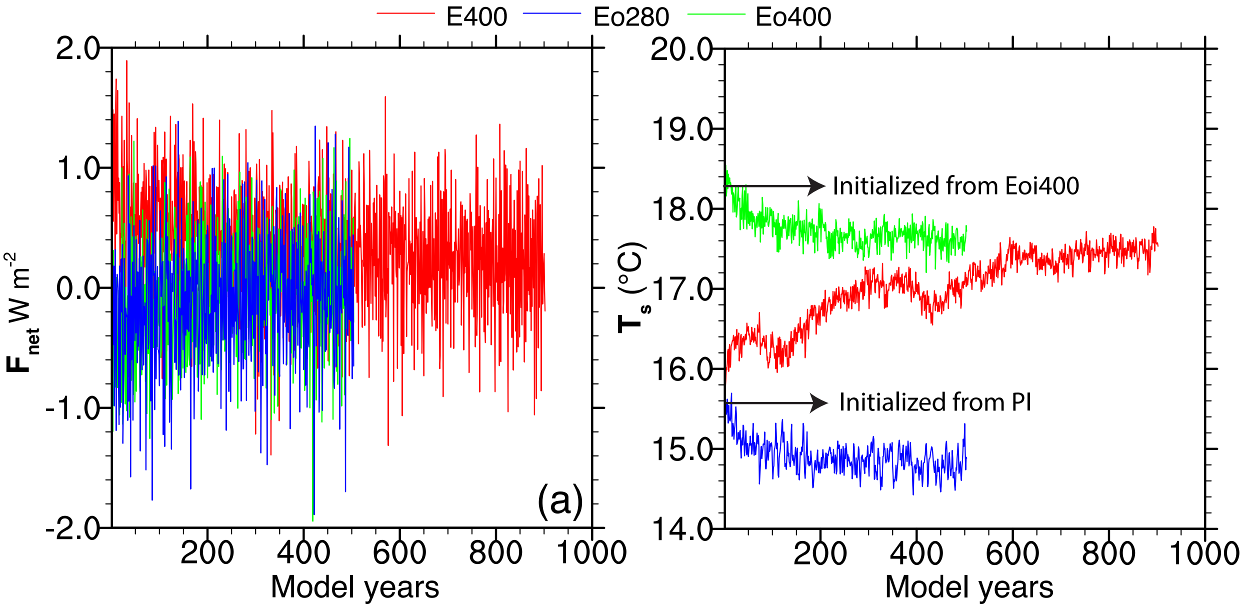

Fig. S13 Time series of global mean net top of the atmosphere radiation imbalance (F_{net}) and surface temperature for the entire simulations of E400, Eo400, and Eo280. To reduce computational cost, Eo400 is initialized from the 1200-year full forcing simulations of the mid-Pliocene (Feng et al., 2020). E400 and Eo280 are both initialized from the preindustrial state. As shown in Fig. S8, influences of initialization or background climate states are minimal for the study regions in the long simulations.

References

1. Pound, M. J., Tindall, J. & Pickering, S. J. Late Pliocene lakes and soils: a global data set for the analysis of climate feedbacks in a warmer world. *Climate of the …* (2014).
2. Fauquette, S. *et al.* Climate and biomes in the West Mediterranean area during the Pliocene. *Palaeogeography, Palaeoclimatology, Palaeoecology* **152,** 15–36 (1999).
3. Blavoux, B., Dubar, M. & Daniel, M. Indices isotopiques (13C et 18O) d'un important refroidissement du climat à la fin du Pliocène (formation lacustre de Puimoisson, Alpes-de-Haute-Provence, France): Isotopic indices (13C and 180) of an imponant cooling at the end of the Pliocene (Puimoisson lacustrine formation, Alpes-de-Haute-Provence, France). *Comptes Rendus de l'Académie des Sciences-Series IIA-Earth and Planetary Science* **329,** 183–188 (1999).
4. van Dam, J. A. Geographic and temporal patterns in the late Neogene (12–3 Ma) aridification of Europe: the use of small mammals as paleoprecipitation proxies. *Palaeogeography, Palaeoclimatology, Palaeoecology* **238,** 190–218 (2006).
5. deMenocal, P. B., African climate change and faunal evolution during the Pliocene–Pleistocene. *Earth and Planetary Science Letters* **220,** 3–24 (2004).
6. Demenocal, P. B. Plio-pleistocene African climate. *Science* **270,** 53–59 (1995).
7. Zazzo, A. *et al.* Herbivore paleodiet and paleoenvironmental changes in Chad during the Pliocene using stable isotope ratios of tooth enamel carbonate. *Paleobiology* **26,** 294–309 (2000).
8. Levin, N. E. Environment and climate of early human evolution. *Annu. Rev. Earth Planet. Sci.* **43,** 405–429 (2015).
9. Feakins, S. J., Demenocal, P. B. & Eglinton, T. I. Biomarker records of late Neogene changes in northeast African vegetation. *Geology* **33,** 977–980 (2005).
10. Lupien, R. L. *et al.* Vegetation change in the Baringo Basin, East Africa across the onset of Northern Hemisphere glaciation 3.3–2.6 Ma. *Palaeogeography, Palaeoclimatology, Palaeoecology* 109426 (2019).
11. Campisano, C. J. & Feibel, C. S. Depositional environments and stratigraphic summary of the Pliocene Hadar formation at Hadar, Afar depression, Ethiopia. *The geology of early humans in the Horn of Africa* **446,** 179–201 (2008).
12. Westover, K. S. *et al.* Diatom paleolimnology of late Pliocene Baringo Basin (Kenya) paleolakes. *Palaeogeography, Palaeoclimatology, Palaeoecology* 109382 (2019).
13. Munoz, A., Ojeda, J. & Sanchez-Valverde, B. Sunspot-like and ENSO/NAO-like periodicities in lacustrinelaminated sediments of the Pliocene Villarroya Basin (La Rioja, Spain). *Journal of Paleolimnology* **27,** 453–463 (2002).
14. Heermance, R. V. *et al.* Climatic and tectonic controls on sedimentation and erosion during the Pliocene–Quaternary in the Qaidam Basin (China). *Bulletin* **125,** 833–856 (2013).
15. Rousseau, D.-D., Parra, I., Cour, P. & Clet, M. Continental climatic changes in Normandy (France) between 3.3 and 2.3 Myr BP. *Palaeogeography, Palaeoclimatology, Palaeoecology* **113,** 373–383 (1995).
16. Sanyal, P., Bhattacharya, S. K., Kumar, R., Ghosh, S. K. & Sangode, S. J. Mio–Pliocene monsoonal record from Himalayan foreland basin (Indian Siwalik) and its relation to vegetational change. *Palaeogeography, Palaeoclimatology, Palaeoecology* **205,** 23–41 (2004).
17. Gaur, R. & Chopra, S. Taphonomy, fauna, environment and ecology of upper Sivaliks (Plio-Pleistocene) near Chandigarh, India. *Nature* **308,** 353–355 (1984).
18. IGARASHI, Y., YOSHIDA, M. & TABATA, H. History of vegetation and climate in the Kathmandu Valley. *Proceedings of the Indian National Science Academy, Part A. Physical sciences* **54,** 550–563 (1988).
19. Xie, S. *et al.* Palaeoclimatic estimates for the late Pliocene based on leaf physiognomy from western Yunnan, China. *Turkish Journal of Earth Sciences* **21,** 251–261 (2012).
20. Wang, J., Wang, Y. J., Liu, Z. C., Li, J. Q. & Xi, P. Cenozoic environmental evolution of the Qaidam Basin and its implications for the uplift of the Tibetan Plateau and the drying of central Asia. *Palaeogeography, Palaeoclimatology, Palaeoecology* **152,** 37–47 (1999).
21. Koutsodendris, A. *et al.* Late Pliocene vegetation turnover on the NE Tibetan Plateau (Central Asia) triggered by early Northern Hemisphere glaciation. *Global and Planetary Change* **180,** 117–125 (2019).
22. Yao, Y.-F. *et al.* Monsoon versus uplift in southwestern China–late Pliocene climate in Yuanmou Basin, Yunnan. *PLoS One* **7,** e37760 (2012).
23. Chang, Z., Xiao, J., Lü, L. & Yao, H. Abrupt shifts in the Indian monsoon during the Pliocene marked by high-resolution terrestrial records from the Yuanmou Basin in southwest China. *Journal of Asian Earth Sciences* **37,** 166–175 (2010).

24. Ji, S., Nie, J., Breecker, D. O., Luo, Z. & Song, Y. Intensified aridity in northern China during the middle Piacenzian warm period. *Journal of Asian Earth Sciences* **147,** 222–225 (2017).
25. Sun, Y., An, Z., Clemens, S. C., Bloemendal, J. & Vandenberghe, J. Seven million years of wind and precipitation variability on the Chinese Loess Plateau. *Earth and Planetary Science Letters* **297,** 525–535 (2010).
26. Lu, H., Wang, X. & Li, L. Aeolian sediment evidence that global cooling has driven late Cenozoic stepwise aridification in central Asia. *Geological Society, London, Special Publications* **342,** 29–44 (2010).
27. Gent, P. R. *et al.* The community climate system model version 4. *J. Climate* **24,** 4973–4991 (2011).
28. Dowsett, H. *et al.* The PRISM4 (mid-Piacenzian) paleoenvironmental reconstruction. *Climate of the Past* **12,** 1519–1538 (2016).
29. Hurrell, J. W. *et al.* The community earth system model: a framework for collaborative research. *Bull. Amer. Meteor. Soc.* **94,** 1339–1360 (2013).
30. Danabasoglu, G. *et al.* The Community Earth System Model version 2 (CESM2). *Journal of Advances in Modeling Earth Systems* **12,** e2019MS001916 (2020).
31. Jungclaus, J. H. *et al.* Ocean circulation and tropical variability in the coupled model ECHAM5/MPI-OM. *J. Climate* **19,** 3952–3972 (2006).
32. Hazeleger, W. *et al.* EC-Earth V2. 2: description and validation of a new seamless earth system prediction model. *Clim Dyn* **39,** 2611–2629 (2012).
33. Gordon, C. *et al.* The simulation of SST, sea ice extents and ocean heat transports in a version of the Hadley Centre coupled model without flux adjustments. *Clim Dyn* **16,** 147–168 (2000).
34. Marti, O. *et al.* Key features of the IPSL ocean atmosphere model and its sensitivity to atmospheric resolution. *Clim Dyn* **34,** 1–26 (2010).
35. Boucher, O. *et al.* Presentation and evaluation of the IPSL-CM6A-LR climate model. *Journal of Advances in Modeling Earth Systems* **12,** e2019MS002010 (2020).
36. Hasumi, H. & Emori, S. K-1 coupled model (MIROC) description. K-1 Technical Report 1. *Center for Climate System Research, University of Tokyo* (2004).
37. Zhang, Z. S. *et al.* Pre-industrial and mid-Pliocene simulations with NorESM-L. *Geoscientific Model Development* **5,** 523–533 (2012).
38. Hewitt, H. T. *et al.* Design and implementation of the infrastructure of HadGEM3: The next-generation Met Office climate modelling system. *Geoscientific Model Development* **4,** 223–253 (2011).
39. Kelley, M. *et al.* GISS-E2. 1: Configurations and climatology. *Journal of Advances in Modeling Earth Systems* **12,** e2019MS002025 (2020).
