# Supplementary material for: Past terrestrial hydroclimate sensitivity controlled by Earth system feedbacks
Source: Nat Commun. 2022 Mar 14;13:1306. doi: 10.1038/s41467-022-28814-7 (PMC8921287; doi:10.1038/s41467-022-28814-7)
Supplement: Supplementary file 3 — Description of Additional Supplementary Files [file 41467_2022_28814_MOESM3_ESM.pdf]

Description of Additional Supplementary Files

File Name: Supplementary Data 1
Description: Mid-Pliocene (3.0 – 3.3 Ma) hydroclimate indicators compiled from published records.
